# Full-length genome sequence of porcine epidemic diarrhea virus strain SDTA13-2020

**DOI:** 10.1128/MRA.00014-23

**Published:** 2023-08-18

**Authors:** Wenying Zhao, Yali Yao, Yunjing Zhang, Baicheng Huang, Kegong Tian

**Affiliations:** 1 National Research Center for Veterinary Medicine, Luoyang, China; Katholieke Universiteit Leuven, Leuven, Belgium

**Keywords:** porcine epidemic diarrhea virus

## Abstract

We report here the complete genome sequence of porcine epidemic diarrhea virus (PEDV) strain SDTA13-2020, isolated from a suckling piglet with watery diarrhea in Shandong, China. The isolate is genetically close to other recent Chinese G2 genotype PEDVs and distinct from the classical PEDVs.

## ANNOUNCEMENT

Porcine epidemic diarrhea virus (PEDV) is an enveloped, positive-sense single-stranded RNA virus belonging to the genus *Alphacoronavirus*, family *Coronaviridae* (1). It is the major causative virus in acute watery diarrhea, vomiting, and dehydration, with high mortality that often reaches 100% in neonatal piglets (2, 3). In 1978, PEDV was first isolated in pig farms in Belgium (4). PEDV first appeared in China in the 1980s (5). Since late 2010, a large-scale PEDV epidemic broke out in Chinese pig herds. Since 2013, highly virulent PEDV variants have been reported in the United States, Canada, Japan, Colombia, and other places (6). Currently, PEDV is divided into three genotypes, G1, G2, and S-INEDL.

In March 2020, the intestinal contents of a suckling piglet with acute diarrhea were collected from a pig farm in Shandong, China. PEDV was identified by an established reverse transcription-PCR with primers specific to the M gene of PEDV (7). Subsequently, the PEDV positive sample was diluted into free-serum Dulbecco’s Modified Eagle Medium (DMEM; Thermo Fisher Scientific, Waltham, MA, USA), then centrifuged at 10,000 × *g* for 10 min at 4°C. The supernatants were collected and filtered through a syringe filter with a 0.22 µm size. Then, 200 µL supernatant was mixed with 200 µL DMEM, including 5 µg/mL trypsin (Sigma-Aldrich, St. Louis, MO, USA), inoculated onto monolayers of Vero cells (American Type Culture Collection, ATCC), and incubated at 37°C, 5% CO_2_ for 1 h. The fourth passage of supernatant was used for RNA preparation. Total RNA was extracted using Viral Nucleic Acid Extraction KitII (Geneaid, Taiwan, China). Primers used for complete genome sequencing were designed based on conserved regions of the full-length sequences of PEDV after multiple-sequence alignment (Table 1). Meanwhile, the 5’ and 3’ terminal ends of the virus genome were amplified using the SMART 5′ RACE and 3′ RACE (Clontech, Japan) and a gene-specific primer (8), respectively, following the manufacturer’s instructions. The PCR amplicons were gel purified and cloned into the *pEasy*-blunt vector (TransGen Biotech, Beijing, China). Three or four clones from each PCR product were sequenced by Sangon Biotech (Shanghai, China). Finally, the data were assembled and annotated by Lasergene version 7.0 with default parameters. The PEDV strain was named SDTA13-2020. The PEDV SDTA13 open reading frames (ORFs) were annotated in SeqBuilder based on a manually curated PEDV database retrieved from GenBank.

**TABLE 1:** Primers used in the complete genome sequence

Primer name	Primer sequence (5′−3′)	Size/bp
seq-1-F	TCTAGACTCTTGTCTACTCAATT	2,062
seq-1-R	CAGGTATTGGGTCACACTC	
seq-2-F	ACTTGCATTCTTCGAGAGTGACG	1,524
seq-2-R	GGATCTTTAGTAACTGTGGAAGGTG	
seq-3-F	GTGATGAAGTAGACTCCTCTG	2,214
seq-3-R	GTAGACCTGCTGACTTGAAC	
seq-4-F	TCCACATGCTAGACCATACTG	2,233
seq-4-R	ATACACGTGGCAATGTCATGGAC	
seq-5-F	ACATGCAGGATTGCAAGAGC	2,138
seq-5-R	GTACTTAGGCGTGTGGACAT	
seq-6-F	ACGCCATGTTATAGCGTCTAG	2,210
seq-6-R	TTGCCAAGTTGAGAATAGTGT	
seq-7-F	ATGGTTCTCCACCTCAGTTG	2,147
seq-7-R	CACAGTGAACTATGCACTGCT	
seq-8-F	ACGGAGCATAAGACAGCACT	2,033
seq-8-R	CGTAGACAATCACCACAACGT	
seq-9-F	ATGCCTACCCGTTATCTAAGC	2,203
seq-9-R	TGATGGCTGGGTATGTTGAT	
seq-10-F	AGCAGAGGTGATGATCTGTTGC	2,209
seq-10-R	CACCATACACAACGTGCTCA	
seq-11-F	CCTGATGGCTATTTTACCCAA	1,656
seq-11-R	CAATCTCAAAGCCATGACCAC	
seq-12-F	ATATCTTCTGGCGTAATTCCAC	2,069
seq-12-R	GGGACGTAAACTTAACACCAC	
seq-13-F	AGTAATTGCCCTTTCACCTTG	1,790
seq-13-R	ACATCTACAAAGTCACCCGGTA	
seq-14-F	ACTTCAGCCTTTGAGAGTGT	1,541
seq-14-R	AGCTGCTTTACCATTGAGGA	
seq-15-F	TGTTGCACACTTATTGGCAGGC	2,106
seq-15-R	TCTTTCGAAGTGGCTCTGGAT	
seq-16-F	ACATCACGCGATGATCTGGT	1,057
seq-16-R	GTGTATCCATATCAACACCGTCAG	
5’RACE-R	GATTACGCCAAGCTTAGTGCCAGCACCAGCGCGCGTAACC	1,174
3’RACE-F	GATTACGCCAAGCTTAATTCGCTGGCGCATGCGCCGTGGT	1,506

The complete genome sequence of SDTA13-2020 was 28,038 bp in length, with a G + C content of 41.74%. The non-coding region and coding regions are arranged on the genome in the order: 5′-UTR (nt1-292), replicase polyprotein (nt293-12646 for 1a and nt12811-20637 for 1b), spike protein (nt20634-24794), ORF 3 (nt24794-25468), envelope protein (nt25687-26367), membrane protein (nt26379-27704), nucleocapsid protein (nt27705-28038), and 3′-UTR (nt27705-28038). Nucleotide similarity analysis was performed by MegAlign software. The complete genome sequence of SDTA13-2020 displayed the nucleotide sequence identities of 98.5%, 97.9%, and 96.6% to vaccine strain AJ1102 (GenBank accession number JX188454.1), vaccine strain LW/L (GenBank accession number MK392335.1), and Belgian classical strain CV777 (GenBank accession number AF353511.1), respectively.

SDTA13-2020 strain belonged to G2 genotype. Interestingly, the SDTA13-2020 strain possessed a four amino acid insertion (^59^QGVN^62^) and a single amino acid insertion (N^139^) compared to the PEDV classical strain CV777 in the S gene, and a single amino acid insertion (H^1193^) compared to AJ1102 and LW/L strains. In addition, PEDV ORF3 gene showed seven variant sites (V21A, V54I, V79I, L92F, A101T, N166S, and H182Q) compared to CV777 strain. The genome information of strain SDTA13-2020 will enable a better understanding of the evolutionary characteristics and molecular epidemiology of PEDV in China.

## Data Availability

The complete genome sequence of PEDV strain SDTA13-2020 has been deposited in GenBank under the accession number OP245917. SRA accession numbers, SRR24124291 and SRR24150200.
